# The effect of clear aligner treatment on masticatory muscles (masseter, temporalis) activity in adults: a systematic review and meta-analysis

**DOI:** 10.1093/ejo/cjae030

**Published:** 2024-06-29

**Authors:** Roberta Lekavičiūtė, Smiltė Paldauskaitė, Simona Stučinskaitė, Giedrė Trakinienė

**Affiliations:** Faculty of Odontology, Lithuanian University of Health Sciences, J. Lukšos-Daumanto Str. 2, Kaunas, Lithuania; Faculty of Odontology, Lithuanian University of Health Sciences, J. Lukšos-Daumanto Str. 2, Kaunas, Lithuania; Private Practice, Kaunas, Lithuania; Department of Orthodontics, Lithuanian University of Health Sciences, J. Lukšos-Daumanto Str. 6, Kaunas, Lithuania

**Keywords:** clear aligners, masticatory muscle activity, mastication, clinical trials, systematic reviews and meta-analyses

## Abstract

**Background:**

The use of clear aligners is becoming more common for aesthetic orthodontic treatment, but there are still concerns about how they affect mastication biomechanics in the short and long term. The clear aligners treatment (CAT) mechanism changes the position of the mandible and maxilla, especially impacting the masseter muscle. Surface electromyography (sEMG) proves to be a useful method to evaluate masticatory muscle activity (MMA).

**Objectives:**

To analyze the effect of clear aligners treatment on alterations in masticatory muscles (masseter, temporalis) using surface electromyography.

**Search methods:**

Five databases (PubMed, Web of Science, SCOPUS, Cochrane Library, and Google Scholar) were searched up to March 2024.

**Selection criteria:**

Studies in which MMA was evaluated after the installation of orthodontic clear aligners.

**Data collection and analysis:**

Screening, data extraction, and quality assessments were performed by four investigators independently. The data, which evaluated temporalis and masseter muscle characteristics during CAT using surface electromyography, was extracted, and the quality of the studies was evaluated. The risk of bias was assessed using the Newcastle-Ottawa Scale (NOS).

**Results:**

Six studies (two prospective cohort studies, three observational longitudinal studies, and one observational longitudinal case–control study) with low and moderate risk of bias were included in the qualitative synthesis. Six of these were also included in the meta-analysis. Our study investigated the dynamics of masseter and temporalis muscle activity during CAT. The results show that during maximal voluntary clenching, the masseter muscle demonstrated a significant initial increase (*P* < .05) followed by a subsequent non-significant decrease. It also showed that submaximal voluntary clenching consistently exhibited a significant reduction in muscle activity throughout the study period (*P* < .01). Assessment of muscle activity at the mandibular resting position revealed a variety of responses, with some participants showing a significant increase while others exhibited non-significant changes (*P* < .05, *P* > .05, respectively). However, the meta-analysis showed a non-significant difference in measuring masseter and temporalis muscles activity during CAT.

**Conclusions:**

Based on existing evidence, it is reasonable to conclude that CAT affected MMA. During maximal voluntary clenching, masseter muscle activity initially increased but later decreased, while temporalis muscle activity showed mixed results. Submaximal voluntary clenching revealed a consistent decrease in muscle activity over time. Mandibular resting position assessments showed both increases and no significant changes in muscle activity. However, the existing literature is insufficient to draw concrete conclusions; therefore, well-conducted further research is needed to confirm this statement.

**Registration:**

This systematic review and meta-analysis were registered in the International Prospective Register of Systematic Reviews (PROSPERO CRD42024522231).

## Introduction

These days, orthodontic treatment with clear aligners is becoming more popular because of the increasing desire for aesthetic and comfortable alternatives to traditional fixed appliances [1]. The relevance of aesthetic values is based on wearing a transparent orthodontic appliance due to the approach to meet society’s current demands [2]. However, there are different opinions about the correlation between clear aligners treatment and masticatory muscle activity [3]. Anyhow, some studies show that orthodontic treatment and appliances can affect masticatory muscle activation [4, 5]. Aligners’ treatment mechanism is to change the maxilla and mandible position, enabling attached muscles to change to a certain extent. This way the masseter muscle changes [6]. The stability of occlusion is connected to neuromuscular function [7].

Standardized surface electromyography (ssEMG) is an objective and useful instrumental approach in the analysis of dental occlusion, which may comprehensively assess bioelectrical muscle activity, measuring their fatigue or neuromuscular coordination [8]. Electrodes are placed on the skin above specific muscles and measure electrical signals of the muscle [7]. Studies show that even a small 0.25 mm aluminum intercuspal interference (in comparison, a set of aligners are usually 1,5 mm), could give rise to asymmetric contractile activity in the mandibular elevator muscles studied by means of ssEMG, as well as potentially displacing the mandible in a lateral direction [9]. Furthermore, ssEMG can be used to determine if a patient needs a retention after orthodontic treatment [10]. To optimize the validity, sensitivity, and reliability of sEMG measurements, however, the processing and interpretation of electromyographic data are intricate and need specific indices [10].

The aim of this literature review with meta-analysis was to evaluate the correlation between orthodontic aligners treatment and alterations in masticatory muscles.

## Methods

### Definition of the research question

This systematic review and meta-analysis investigate the correlation between clear aligners treatment in adults (mean ages 22–35). The developed focus question was: Does clear aligners treatment affect masticatory muscle activity in adults?

### Registration and protocol

These systematic reviews and meta-analyses were conducted and reported according to updated guidelines of Preferred Reporting Items for Systematic Reviews and Meta Analysis (PRISMA) 2020 statement [11], and the protocol was registered in the International Prospective Register of Systematic Reviews (PROSPERO CRD42024522231).

### Eligibility criteria

For the development of the question focus, the PICO (Patient/Problem, Intervention, Comparison, Outcome) study design protocol was used. Clinical trials (S) performed in human adults of any sex (P), undergoing orthodontic treatment with clear aligners (I), comparing masticatory muscle activity (C). The outcomes (O) of this systematic review included following treatment masseter and temporalis muscle conditions.

### Inclusion criteria

- All published study designs published in English except case reports and literature reviews
- Research on patients undergoing orthodontic clear aligners treatment
- Clinical studies regarding the impact of clear aligners treatment on masticatory muscle activity
- Studies that used sEMG to evaluate alterations in masticatory muscles
- Studies using clear aligners of a 0.7–0.8 mm thickness

### Exclusion criteria

- Literature reviews, meta-analyses, studies reporting data from case reports and series, studies on animals, and manuscripts that did not state the names of the authors
- Articles that do not evaluate clear aligners treatment association with masticatory muscle activity
- Researches that analyze masticatory muscle changes but clear aligners treatment are not considered
- Clinical studies that include patients undergoing clear aligners treatment but do not analyze masticatory muscle activity
- Studies that did not use sEMG to evaluate alterations in masticatory muscles
- Patients with genetic syndromes (craniofacial syndromes, cleft lip, or palate), severe facial malformations or systemic diseases

### Information sources and search strategy

The electronic literature searches were performed in the electronic databases PubMed, Web of Science, SCOPUS, Cochrane Library, and Google Scholar up to March 2024 with no publication date restrictions. Databases were systematically searched by two authors using different combinations of the following keywords: (‘clear aligners’ OR ‘orthodontic aligners’) AND (‘masticatory muscle’ OR ‘muscle of mastication’ OR ‘electromyography’). Language restrictions were applied (only articles in English were considered eligible). In addition, all the references of relevant reviews and eligible articles that our search retrieved were checked for any additional trials. Three investigators, working independently, searched the literature and extracted data from each eligible study.

### Study selection and data extraction

The search strategy was discussed by four authors before initiating the search in the selected databases. To choose the papers for a full reading, the article titles and abstracts were the first considerations in the selection process; duplicates were removed. To get a final conclusion, the complete article was read to see if it met the inclusion criteria. Following the reading, the articles that were to be included or excluded were screened, and each article’s exclusion reason was noted. The entire process was carried out by three investigators (R.L., S.P., and S.S.), each working independently. If there was any debate about whether an article qualified or not, a fourth author (G.T.) mediated the discussion until an agreement was reached.

Three authors independently extracted study characteristics and outcomes, with the potential disagreements solved by a fourth author (G.T.). For each study, the following information was included: author and year of publication, study design, sample size, masticatory muscle characteristics, and their activity measurements.

### Risk of bias within studies

To assess the quality of studies, the Newcastle-Ottawa Scale (NOS) was used. Each study was reviewed by three authors independently, and any disagreements were solved by discussion to reach an agreement.

### Synthesis of results

A meta-analysis was conducted on the quantitative data using open-access RStudio software, Version 2023.12.1 + 402 (2023.12.1 + 402). A Random-Effects Model was run in order to estimate the true effect of clear aligners on the changes in masseter and temporalis muscles sEMG activity. The Random-Effects model was chosen over the Fixed-Effects model because it better incorporates between-study variance, makes results more generalizable beyond the specific studies included, and is suitable for small studies as it avoids overly precise conclusions. However, it is worth acknowledging that compared to the Fixed-Effects model Random-Effects model has some limitations, such as wider confidence intervals due to increased variance. Heterogeneity analysis has been performed using a Random-Effects model. Tau^2^ estimates the actual variance in true effect sizes. I^2^ expresses the proportion (percentage) of variability due to heterogeneity. Q-statistic estimates the significance of heterogeneity. Heterogeneity analysis and model summary are presented in Table 1. The summary effect size estimate has been shown with a Forest Plot. It mainly shows the effect sizes of individual studies (position of the square), sample sizes (size of the square), and confidence intervals (horizontal lines). Small study bias and publication bias have been measured using a funnel plot and Egger’s regression test. Test for effect moderators and confounders has been performed, testing the influence of the mean age, study quality, and study design.

**Table 1.** Heterogeneity analysis.

	Masseter	Conclusion	Temporalis	Conclusion
Tau^2^	0.0129 (CI: 0.00–0.5793)	Relatively small variance	0.0570 (CI: 0.00–0.8119)	Relatively small variance
I^2^	14.99% (CI: 0.00–89.4523%)	Low heterogeneity	45.53% (CI: 0.00–92.25%)	Low heterogeneity
Q-statistic (df = 5)	6.49 (*P* = .2614)	Keep null hypothesis of no heterogeneity	7,1787 (*P* = .1267)	Keep null hypothesis of no heterogeneity

### Sensitivity analysis

Sensitivity analysis has been performed to measure the impact removing a single observation on the various statistics of the model. It identifies influential observation, i.e. whether any observation pulls the overall result in its direction. The results of sensitivity analysis for both masseter and temporalis are presented in Table 2.

**Table 2.** Summary of results of sensitivity analysis for masseter and temporalis muscles.

		rstudent	dffits	cook.d	cov.r	tau2.del	QE.del	hat	weight	dfbs
**M**	Nota A.	−0.737	−0.360	0.150	1.405	0.026	5.842	0.149	14.978	−0.355
	Lou T.	1.7138	0.7817	0.5228	0.9874	0.0000	3.5528	0.1596	15.9640	0.7919 *
	Dellavia, C.P.B.	−0.9352	−0.4261	0.1981	1.3091	0.0202	5.4695	0.1498	14.9781	−0.4226
	Paes-Souza S.D.A.	1.3799	0.4314	0.1745	0.9424	0.0024	4.5474	0.0860	8.6028	0.4485
	Liu P.	0.0892	−0.0258	0.0010	1.7581	0.0422	6.4491	0.2148	21.4772	−0.0266
	Tepedino M.	−0.8530	−0.4890	0.2820	1.4751	0.0220	5.4703	0.2400	23.9997	−0.4985
**T**	Nota A	−0.5627	−0.2718	0.0856	1.4534	0.0781	6.5071	0.1903	19.0307	−0.2701
	Dellavia, C.P.B	−0.9139	−0.4434	0.2032	1.2818	0.0615	5.6418	0.1903	19.0307	−0.4426
	Paes-Souza S.D.A.	0.4330	0.1805	0.0358	1.3408	0.0765	6.9266	0.1275	12.7516	0.1756
	Liu P.	2.4563	1.4788	1.02221	0.7008	0.000	1.1453	0.2382	23.8202	1.4069*
	Tepedino M.	−0.5276	−0.2889	0.1051	1.6048	0.0830	6.2132	0.2537	25.3667	−0.2943

M: masseter muscle, T: temporalis muscle. *P < 0.05. Bold values in text means that it is statistically significant result (*P* < 0.05).

Measures of influence:

- rstudent: Studentized residuals standardized by the square root of their leverage.Identifies potential outliers based on standardized residuals with high leverage.
- dffits: Change in fitted values when an observation is removed. Large dffits indicate observations pulling predictions in their direction.
- cook.d: Cook’s distance, measures overall influence on multiple parameters. Higher values indicate more influential observations.
- cov.r: Leverage-adjusted covariance ratio. High values suggest observations with less precise parameter estimates if removed.
- tau2.del: Leave-one-out estimates of heterogeneity (tau^2) when removing an observation in a mixed-effects model.
- QE.del: Leave-one-out values of the test statistic for heterogeneity in a mixed-effects model.

Other relevant measures:

- hat: Leverage, measures how much an observation contributes to its own fitted value. High hat values indicate high leverage.
- weight: Weight assigned to an observation in the analysis. Lower weights indicate less influence on the model.
- dfbeta: Change in regression coefficients when an observation is removed. Large dfbeta values indicate observations impacting specific coefficients.

## Results

### Study selection

**Figure 1.**
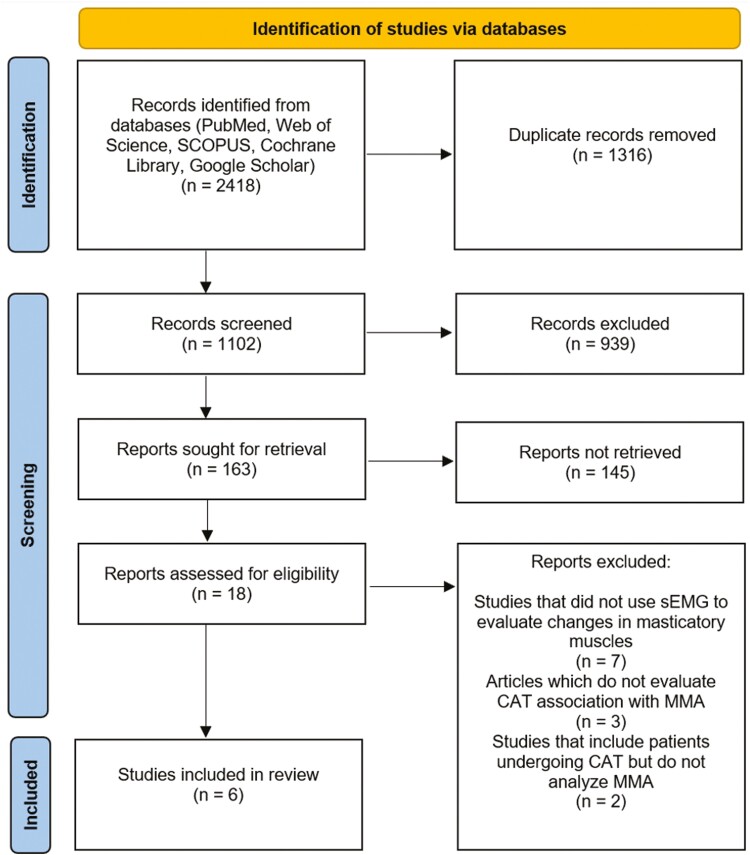
A PRISMA flowchart outlining the study selection process. PRISMA: Preferred Reporting Items for Systematic Reviews and Meta-Analyses; CAT: clear aligners treatment, MMA: masticatory muscle activity, sEMG: surface electromyography

A total of 2418 articles were identified through the online search engine. After the removal of duplicates, articles underwent screening based on their title and abstract. Therefore, 1102 titles or abstracts of articles were evaluated. Following the application of inclusion criteria to the articles, 163 records remained suitable for screening. Furthermore, a total of 145 records were eliminated from the screening process since they were deemed irrelevant to the topic. The full-text analysis was assessed for 18 articles to determine their eligibility for inclusion in the study. After the full-text analysis, 12 articles were excluded based on eligibility criteria. In the end, a total of six studies were included in the review. Figure 1 provides a visual depiction of the search technique and the synthesis of results. The meta-analysis included a total of six studies [12–17].

### Study characteristics

Two of the included studies were prospective cohort studies [12, 13], three observational longitudinal studies [14–16], and one observational longitudinal case-control study [17]. Measurements of the sEMG masseter muscle (MM) activity were reported in six articles [12–17]. In five studies, the recruitment of masseter muscles was evaluated bilaterally [12, 14–17], while one article evaluated activity only in the right masseter muscle [15]. Each of the studies assessed the masticatory muscle activity during maximal voluntary clenching (MVC) [12–17]. Three studies conducted measurements with the mandible in the resting position [12, 14, 16], and only one study evaluated MMA during submaximal voluntary clenching (SMVC) [14]. Only one study analyzed MMA after the end of treatment and also compared the effect of fixed orthodontic appliances to clear aligners on MMA [17]. Synthesis and analysis of findings (PICO) on the investigation are represented in Table 3. On average, each study included approximately 18 patients (ranging from a minimum of 10 to a maximum of 26 patients). The studies involved a total of 108 subjects, with the mean age ranging from 22 to 35 years. Ethical approval was received for all studies from their ethical committees or review boards [12–17].

**Table 3:** Included studies on the investigation of the correlation between MMA and CAT.

Study	Population	Intervention	Comparison	Outcomes
Nota *et al*. [12], 2021	*N* = 16; one group: patients undergoing CAT (mean age 22.5 ± 3.5 years)	sEMG	Activity of the masseter and temporalis muscles	No noticeable changes were detected in the sEMG activity of the TA muscle over the course of the study. Following one month of CAT, participants exhibited a notable decrease in the sEMG activity of the MM during mandibular RP compared to pretreatment measurements. No differences were noted in the activity of both the MM and TA muscles during MVC.
Tepedino *et al*. [13], 2023	*N* = 26; one group: patients undergoing CAT (mean age 33.67 ± 13.33 years)	sEMG	Activity of masseter and temporalis muscles	During MVC MM and TA muscles did not show significant differences in TC, AC, and Asym values. However, there was a significant difference in terms of POC in both MM and TA after 6 months of treatment, while after 3 months no differences were observed.
Paes-Souza *et al*. [14], 2023	*N* = 10; one group: patients undergoing CAT (mean age 29.9 ± 5.5 years)	sEMG	Activity of the masseter and temporalis muscles	It was found that MM activity increased from 1 to 2 weeks but was followed by a decrease over time resulting in a ~20% increase at the end of 32 weeks during MVC. Furthermore, during MVC TA activity significantly increased from the beginning to 1–4 months of CAT, resulting in ~70% increase at the end of the following period. In MM sEMG signal amplitude in SMVC decreased by 20 % from pretreatment to the end of follow-up period. While analyzing TA activity during SMVC a significant increase was noticed from the beginning of CAT to the 1–4 months of treatment. The TA muscle recruitment significantly increased at the end of the follow-up period (90%). While mandible was at the RP a significant increase was noticed in MM activity at the end of evaluation period (~30 %). During mandible RP TA muscle activity increased from one week before CAT to 4-32 weeks of aligners, resulting in the 80 % increase of muscle recruitment at the end of the period.
Lou *et al*. [15], 2020	*N* = 17; one group: patients undergoing CAT (mean age 35.3 ± 17.6 years)	sEMG	Activity of the right masseter muscle	The MM activity significantly increased when wearing aligners compared to the baseline. The greatest increase in sEMG activity relative to the baseline was observed during week 2 with a passive aligner and week 3 with the first active aligner, while the smallest increase occurred during week 4 with the second active aligner. No disparity in sEMG activity was noted between the passive aligner and the first active aligner. However, there was a significant decrease in MM activity from the first to the second active aligner.
Liu et al. [16], 2017	N = 23; one group: patients undergoing CAT (mean age 26.8 ± 2.4 years)	sEMG	Activity of masseter and temporalis muscles	During MVC there was no statistical difference in MM before and during CAT. However, after 3 months of the CAT there was a significant difference in TA, but no differences were seen before and after 6 months of the treatment. While mandible was at RP no changes were observed in MM before CAT, after 3 and 6 months of CAT, but TA showed significant increase after 3 months of the treatment.
Dellavia *et al*. [17], 2022	*N* = 16; 2 groups: patients with fixed orthodontic appliances (*n* = 7), undergoing CAT (*n* = 9) (mean age 25.60 ± 13.17 years)	sEMG	Activity of the masseter and temporalis muscles	Before CAT, TC, AC, Asym, and POC values of MM were within the normal range, while at the end of CAT, patients presented an increase in POC and in AC values. However, no significant differences were found in TC and Asym values. Furthermore, after 3 months of retention, the mean values of each parameter were within the normal range. AC, TC, and POC values of TA muscle were within the normal range at the beginning and end of CAT, with stable POC TA and TC. Three months after treatment, the mean values of each parameter were within the normal range.

### Methodological quality assessment of included studies

**Figure 2.**
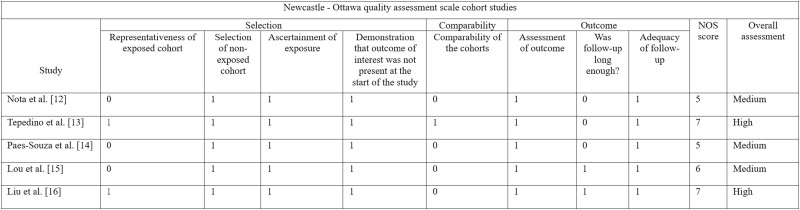
Quality assessment of the included cohort studies according to Newcastle-Ottawa Scale (NOS).

**Figure 3.**
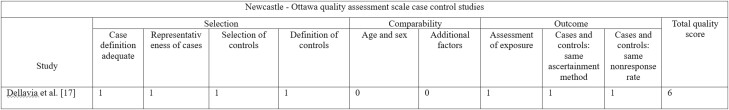
Quality assessment of the included case–control studies according to Newcastle-Ottawa Scale (NOS).

The NOS risk of bias assessment tool was used to evaluate the methodological quality of five cohorts and one case–control studies. Three domains were considered: selection, comparability, and outcome. In case of disagreement between reviewers, a mutual decision through discussion was made (Figs 2–3).

### Results of individual studies

The outcomes of all individual studies are summarized in Table 3, which presents MMA measurements with the mandible in the resting position, during maximal and submaximal voluntary clenching.

### Changes in masticatory muscle activity observed before, during, and after treatment

All the studies measured the activity of masticatory muscles before orthodontic treatment with clear aligners and compared it with values at different treatment stages [12–17]. In one study, there was a significant increase in the sEMG activity of the masseter muscle during the first week with a passive aligner and another increase during the first week with the active aligner (*P* < .001). Furthermore, a significant decrease towards baseline levels in the activity of the masseter was observed after one month of treatment [15]. MMA was also measured after one month of treatment in another study and showed a significant reduction in masseter activity during the mandibular rest position; however, the anterior temporalis muscle activity showed no significant changes [12]. Two studies compared muscle activity after three and six months of treatment [13, 16]. The sEMG of the temporalis anterior after three months of treatment was significantly higher than at baseline (*P* < .05). Subsequently, it slightly decreased from 3 to 6 months, with the value at 6 months still higher than before the treatment (*P* > .05). However, another study found no difference in the activity of the masseter muscle among all time points [16]. Symmetry in the activation of muscular couples remained consistent over the 6-month follow-up period [13]. The activity of both the superficial masseter and anterior temporal muscles was measured up to 8 months of treatment. The sEMG activity significantly increased at the end of the evaluation period (*P* = .001) and continued to rise throughout the treatment during rest conditions [14]. Only one study analyzed how masseter and anterior temporalis muscle activity changed after the end of treatment and found that the mean values of each parameter returned to the normal range three months after the removal of the last aligner [14].

### Masseter muscle parameters

#### Measurements during maximal voluntary clenching

Two of the included studies showed that MM activity increased at the beginning of CAT but was followed by a decrease over time [14, 15]. In the study done by Lou *et al*., the biggest significant increase in MM activity occurred while using the passive aligners (week 2) and the first active aligners (week 3) compared to week 1 without aligners (*P* < .001). However, MM activity significantly decreased between the first and second active aligners (week 4) (*P* < .001) [13]. On the other hand, the least significant increase was with the second active aligners (*P* < .001), although there was no statistically significant difference in sEMG activity between week 1 and week 2 (*P* = .751) [13]. Another research conducted by Paes-Souza *et al*. found that RMS amplitude increased from 1 to 2 weeks of CAT but was followed by a gradual decrease over time, resulting in a ~20% increase toward the end at 32 weeks [14].

Nota *et al*. [12] and Liu *et al*. [16] stated that sEMG of MM showed no difference between before CAT and during treatment. One study evaluated MM activity before CAT, 1 month, and 3 months of treatment [12], while another before CAT, 3 months, and 6 months of treatment [16].

Assessments of Percentage Overlapping Coefficient (POC), Torque Coefficient (TC), Activity Index (Ac), and Asymmetry Index (Asym) of MM were evaluated in two studies [13, 17]. Both investigations revealed significant alterations in POC [13, 17], with only one study demonstrating significant changes in Ac [17]. However, neither study indicated significant differences in TC or Asym [13, 17]. Dellavia *et al*. investigated the MM activity before the beginning of CAT (T1), at the end (T2), and 3 months (T3) after the end of the treatment [17]. At T1, TC, AC, Asym, and POC values were within the normal range, while at the T2 timepoint, patients presented an increase in POC (from 83.2% at T1 to 86.0% at T2) and in AC values (from 3.9% at T1 to 6.8% at T2) (*P* < .05 for both). However, no significant differences were found in TC and Asym values (*P* > .05 for both). Moreover, only in this study, MMA was analyzed 3 months after the end of treatment (T3), the mean values of each parameter were within the normal range [17]. Tepedino *et al*. research did not reveal any statistically significant differences in TC, AC, and Asym values, but statistically significant differences were observed in terms of POC for the masseter muscle after 6 months after the start of treatment (*P* < .05), however, there was no significant difference after 3 months (*P* > .05) [13].

#### Measurements during submaximal voluntary clenching

Only one study conducted by Paes-Souza *et al*. evaluated the MM activity during SMVC [14]. The root mean square (RMS) of the sEMG signal amplitude in SMVC decreased by 20% from pretreatment, one week before using the first pair of aligners (T0), to the end of follow-up, when 32 weeks have passed since the first pair of aligners were installed (T8). At the time of T0, the right masseter’s RMS was 108.6 ± 40.8%, exhibiting a decrease to 88.7 ± 51.3% by T8, while the left masseter’s RMS shifted from 87.8 ± 20.8% at T0 to 68.1 ± 23.0% [14].

#### Measurements with the mandible in the resting position

Three articles [12, 14, 16] assessed the masseter muscle activity during measurements with the mandible at rest position (RP). Two studies reported significant changes in the surface electromyographic activity of the masseter muscle [12, 14]. Nota *et al*. evaluated MM activity at the initial phases of orthodontic treatment with clear aligners and stated that after 1 month of treatment using clear aligners and reported a significant decrease in patients’ MM activity after one month of clear aligners treatment compared to before the start of orthodontic treatment (*P* < .05) [12]. Paes-Souza *et al*. investigated bilateral recruitment of superficial masseter during the 8-month follow-up and reported a significant increase at the end of the evaluation period, with an approximately 30% rise in the sEMG root mean square (RMS) value (*P* = .001), and this heightened activity was sustained throughout the treatment [14]. One of the studies did not reveal a significant change in the sEMG of MM. Liu *et al*. presented the conclusion that the electromyographic activity of the masseter muscle exhibited no significant changes before wearing the aligner, after 3 months, and 6 months of treatment (*P* > .05) [16].

### Temporalis muscle parameters

#### Measurements during maximal voluntary clenching

Measurements of the sEMG temporalis muscle activity (TMA) during MVC were reported in five articles [12–14, 16, 17].

Two of the studies found a statistically significant increase in anterior temporalis muscle (TA) activity during CAT [14, 16]. Paes-Souza *et al*. stated that RMS amplitude significantly increased, progressively from the beginning of CAT to 1–4 months of treatment (*P* = .000). Furthermore, at the end of the follow-up period, muscle recruitment increased by about 70% [14]. Another study done by Liu *et al*. found that the sEMG activity of TA significantly increased after 3 months of CAT compared to before the treatment [16]. However, in one of the studies conducted by Nota *et al*., there was no statistically significant difference observed in the activity of the TA muscle over time (*P* > .05) [12].

In two of the studies, POC, TA, TC, and the Asym of TA were measured [13, 17]. Both studies demonstrated significant changes in POC. Dellavia *et al*. found that AC, TC, and POC values were within the normal range at the beginning and end of CAT, with stable POC TA and TC, except for two patients showing slightly lower POC TA. Only in this study effect of CAT on MMA was analyzed 3 months after the end of the treatment, the mean values of each parameter were within the normal range, except for two patients exhibiting slightly lower POC TA and one demonstrating a high AC value. Asym was slightly high in one patient and high in another [17]. Tepedino *et al*.’s research also showed significant differences were observed in terms of POC after 6 months of treatment (T2) (*P* < .05), whereas no significant difference was found after 3 months (T1) (*P* > .05) [13]. However, no statistically significant differences were observed in TC, AC, and Asym values [13].

#### Measurements during submaximal voluntary clenching

Paes-Souza *et al*. conducted the only study evaluating temporalis muscle activity during SMVC [14]. The study revealed a significant increase in the RMS amplitude for the TA muscle (*P* = .000), progressively increasing from the beginning of CAT to the 1–4 months mark of treatment. Additionally, muscle recruitment demonstrated a significant increase by the end of the follow-up period (90%). The RMS for the right temporalis muscle at the start of CAT was 140.4 ± 118.9, increasing to 204.7 ± 178.9 after 4 months of treatment, while the left temporalis muscle RMS increased from 86.8 ± 17.1 to 143.2 ± 68.0 [14].

#### Measurements with the mandible in the resting position

Temporalis muscle activity was evaluated with the mandible at rest position in three studies [12, 14, 16]. Significant changes in the sEMG activity of the temporalis muscle were observed in two studies [14, 16]. In the study by Paes-Souza *et al*. temporalis muscle activity during mandibular rest position exhibited a significant increase in the RMS amplitude (*P* = .000), progressively from one week before clear aligners treatment to 4–32 weeks of using aligners. By the end of the observational period, there was an increase in muscle recruitment for the mandibular rest task (80%) [14]. Liu *et al*. [16] showed a significant increase in the electromyographic activity of temporalis muscle after 3 months of CAT compared to before the treatment (28.3 ± 6.5 vs. 23.8 ± 11.7, *P* < .05) [16]. Eventually, there was a slight decrease in sEMG from 3 months to 6 months of CAT, with the 6-month value remaining higher than that of 3 months (*P* > .05) [16]. However, no significant difference was observed in the sEMG activity of the temporalis muscle over time in research conducted by Nota *et al*. [12].

### Masticatory muscle activity: clear aligners treatment compared to traditional brackets therapy

One study conducted by Dellavia *et al*. examined changes in the activity of the masseter and temporalis muscles in patients undergoing orthodontic treatment with clear aligners and compared the results with patients undergoing treatment with fixed orthodontic appliances (FOA) [17]. MMA was measured before, at the end of treatment, and 3 months after treatment. At the beginning of treatment and immediately after the removal of either the brackets or the final aligner, the mean values remained within the normal range for both groups, except for the mean ACTIV value, which slightly exceeded the upper normal limit (15.1%) at the end of treatment in FOA group. However, during follow-up period, after three months of nocturnal use of mobile retainers, the mean values for each parameter were within the normal range for both groups [17]. Furthermore, significant changes were found in POC values of the masseter muscle in the FOA group. The POC values significantly increased at the follow-up compared to both before and at the end of treatment (*P* < .05). On the other hand, in the CAT group, no significant changes were noted between each timepoint [17].

### Results of meta-analysis

Heterogeneity analysis showed that all three measurements indicated a low heterogeneity, making the dataset suitable for further meta-analysis. The model result for masseter was 0.2034 and *P*-value was .0781, while the model result for temporalis was 0.2430 and *P*-value was .1280, both model results demonstrating no significant association.

**Figure 4.**
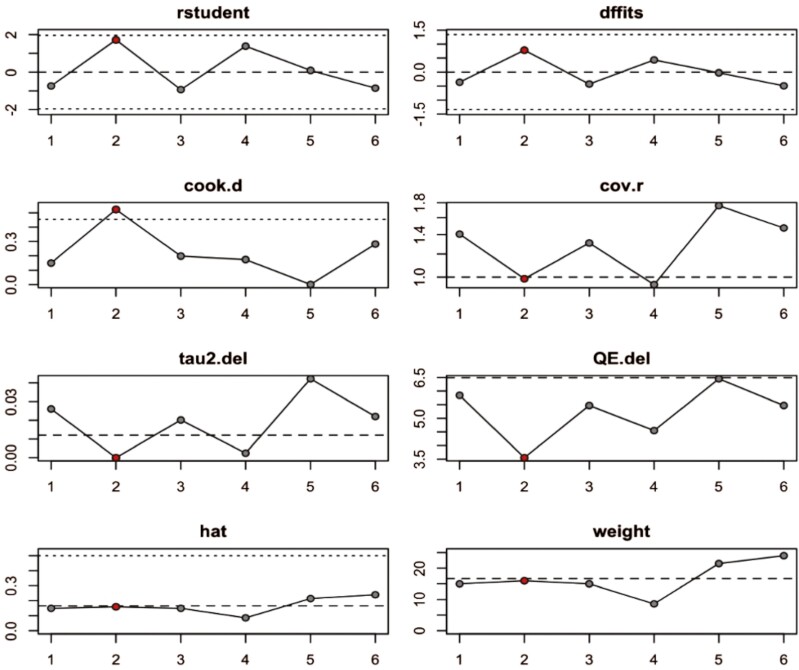
Sensitivity analysis results for masseter muscle.

**Figure 5.**
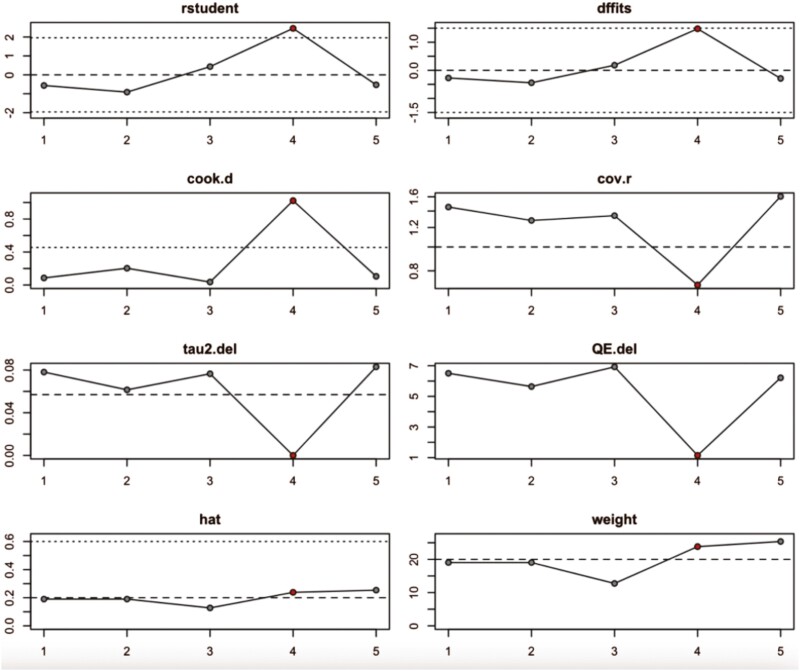
Sensitivity analysis results for temporalis muscle.

Sensitivity analysis results show that Lou *et al*. study has significantly(*) influenced the model of the masseter muscle and Liu *et al*. study has significantly(*) influenced the model of the temporalis muscle. Sensitivity analysis results for the masseter muscle and temporalis muscle are presented in Figs 4 and 5, respectively. Studies that have shown a significant influence on the overall model are shown in red.

**Figure 6.**
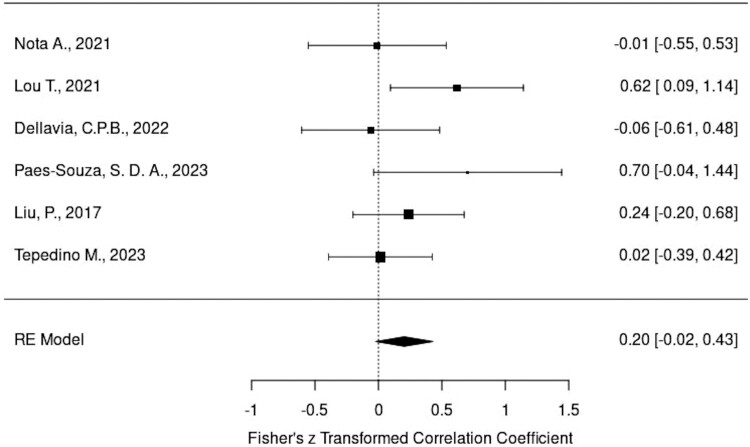
Forest plots of the masseter muscle.

**Figure 7.**
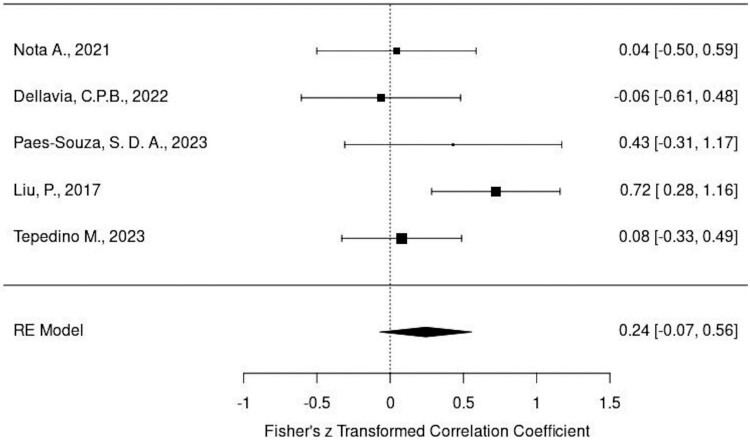
Forest plots of the temporalis muscle.

Forest plots of the masseter muscle and temporalis muscle have been shown in Figs 6 and 7, respectively. The effects size of the masseter muscle was 0.20 and of the temporalis muscle it was 0.24; however, since both confidence intervals (CI) contained 0, it is not considered significant.

**Figure 8.**
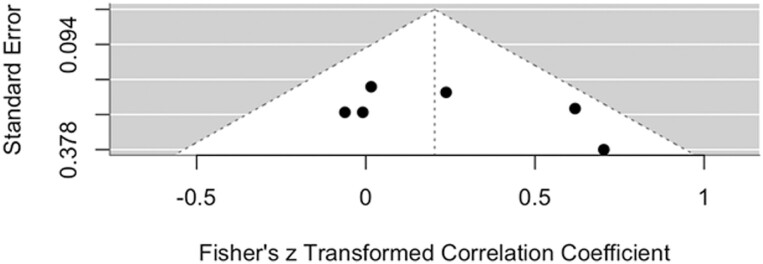
Funnel plot of the studies on masseter muscle.

**Figure 9.**
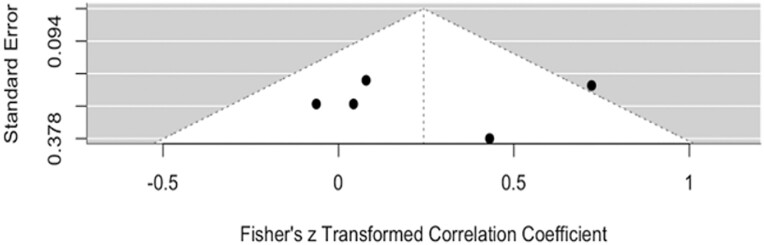
Funnel plot of the studies on temporalis muscle.

Egger’s test for publication bias has been found to be 1.1824 for masseter and −0.0646 for temporalis, with *P* = .237 and *P* = .9485, respectively, indicating that no significant small study bias or publication bias has been detected. The funnel plot for visual inspection of small study bias and publication bias of masseter and temporalis have been shown in Figs 8 and 9, respectively.

Test for effect moderators and confounders has been performed, testing the influence of the mean age, study quality, and study design. None of the moderators in the model have had a moderating effect on the result. Model results are summarized in Table 4.

**Table 4.** Summary of test for effect moderators and confounders results.

		Masseter	Temporalis
**Mean age**	Test of moderators coefficient	0.028	0.0043
	*P*-value	.2835	.9254
**Study quality**	Test of moderators coefficient	−0.260	−0.0239
	*P*-value	.153	.9227
**Study design**	Test of moderators coefficient	−0.3177	−0.3245
	*P*-value	.139	.2969

## Discussion

The present study evaluated alterations of the masseter and temporalis muscle when undergoing clear aligners treatment. Masticatory muscles influence the growth of the jaw, orofacial harmony, and stability [18]. The force of contraction can be recorded by electromyographic activity, which has a direct relationship to tension in the muscle during isometric tasks [19]. Surface electromyography helps to measure muscle activity and record action potentials [16]. Occlusal stability is directly related to neuromuscular adaptation [20]. Muscle imbalance can affect the final result after orthodontic treatment and relapse can occur which requires a prolong lifetime of retainers [21].

In the present study, it was found that MM activity during maximal voluntary clenching is increasing at the beginning of CAT and decreasing overtime [14, 15] or there were no significant changes in MM activity [12, 16]. The disagreement in opinions could be because of a small sample number and different monitoring time. Dellavia *et al*. [17] also compared fixed orthodontic appliances (FOA) to clear aligners and conducted that, for subjects who wore FOA, a slight alteration of the muscular activity appeared immediately after bracket removal and returned in the normal range after 3 months of rescue. In comparison, another study with different bracket systems showed no significant difference in MM activity during clenching [22]. Also, a scientific publication shows an increase in MM activity using clear thermoplastic or wrap-around retainers after orthodontic treatment after 6 months of follow-up [23].

Data on the MM activity when the mandible is at rest is controversial. Nota *et al*. discovered a significant decrease in patients’ MM activity after one month of clear aligners treatment compared to before the start of orthodontic treatment [12]. This reduction can be explained: even a small change in the position of the mandible causes an altered modulation of the masseter muscle [6]. The presence of the appliance could have caused an early and temporary effect on the proprioceptive information given to the central nervous system (CNS) [24]. However, another publication found a significant increase in bilateral recruitment of superficial masseter during the 8-month follow-up. A third opinion was described in Liu *et al*. study [16]. They did not find any alterations of MM activity during and after orthodontic treatment with clear aligners. Regarding functional orthodontic appliances, there is a study with Bimler apparatus [25]. Researchers claim that the subjects wearing the Bimler device constructed in accordance with the neuromuscular (NM) technique exhibited a statistically significant reduction in electromyographic activity of masseter muscle at rest after 6 months of monitoring.

In this study, it was conducted that temporalis muscle activity in maximal voluntary clenching is increasing by two articles [14, 16]. Paes-Souza and coauthors found that TA progressively increased until 4 months of follow-up [14]. Similar data was monitored by Liu *et al*. [16]. TA in maximal voluntary clenching increased after 3 months of CAT. On the other hand, in one study, there was not found any significant changes in TA during CAT. Therefore, a study that involved orthodontic treatment with different bracket systems showed results of TA increasing during clenching [22]. In addition, TA tended to increase during MVC using clear thermoplastic or wrap-around retainers after orthodontic treatment after 6 months of follow-up [23].

When talking about temporalis muscle activity when the mandible is at rest the collected data is resembling maximal voluntary clenching data. Paes-Souza states that TA progressively increased until the 32nd week of CAT [14]. Liu *et al*. found TA enhancement after 3 months of CAT followed by a slight decrease from 3 to 6 months [16]. Nota *et al*. found no TA changes during orthodontic treatment with aligners when the mandible is at rest. In contrast, the subjects wearing the Bimler functional device constructed in accordance with the NM technique showed a statistically significant reduction in TA at rest after 6 months follow-up [25]. Also, the reduction of TA was seen when wearing clear thermoplastic or wrap-around retainers during the observational period of 6 months after orthodontic treatment [23].

This study has some limitations. It is known that surface electromyography results can be inaccurate because sometimes electrodes can pick up activity from nearby muscles. However, sEMG is usually effectively recording masseter and temporalis muscle activity [19]. Also, the studies that were analyzed have rather small samples, different monitoring periods and it was not clear if the studies used the same aligners system (mechanical properties of CA material affect orthodontic forces) [12]. Further studies with larger sample sizes are needed to prepare a more reliable review and to deeply understand neuromuscular responses during orthodontic treatment with CA.

## Conclusions

After analyzing the articles, the following conclusions can be drawn: during maximal voluntary clenching, masseter muscle activity initially increased but later decreased, while temporalis muscle activity showed mixed results; submaximal voluntary clenching revealed a consistent decrease in muscle activity over time; mandibular resting position assessments showed both increases and no significant changes in muscle activity. The performed meta-analysis indicated that although there is an increase in sEMG measurements of the masseter and temporalis muscles during the MVC between before and after the clear aligner treatment, the overall effect size is found to be statistically insignificant, due to the confidence interval containing 0. The findings underscore the complexity of muscular responses to CAT, the results remain controversial, and further research is needed for more reliable conclusions.

## Data Availability

The authors verify that the data supporting the conclusions of this study are accessible within the article or its supplementary materials.
